# Genomewide high-density SNP linkage analysis of non-BRCA1/2 breast cancer families identifies various candidate regions and has greater power than microsatellite studies

**DOI:** 10.1186/1471-2164-8-299

**Published:** 2007-08-30

**Authors:** Anna Gonzalez-Neira, Juan Manuel Rosa-Rosa, Ana Osorio, Emilio Gonzalez, Melissa Southey, Olga Sinilnikova, Henry Lynch, Rogier A Oldenburg, Christi J van Asperen, Nicoline Hoogerbrugge, Guillermo Pita, Peter Devilee, David Goldgar, Javier Benitez

**Affiliations:** 1 Genotyping Unit. CeGen. Human Cancer Genetics Programme, Spanish National Cancer Centre, Spain; 2 Human Genetics Group. Human Cancer Genetics Programme, Spanish National Cancer Centre, Spain; 3 Genetic Cancer Susceptibility Group, IARC, Lyon, France; 4 Plate-forme Mixte de Genetique Constitutionnelle des Cancers Frequents, Hospices Civils de Lyon/Centre Leon Berard, Lyon, France; 5 Creighton University, Omaha, Nebraska, USA; 6 Dept. Of Clinical Genetics, Leiden University Medical Center, Leiden, The Netherlands; 7 Dept. Of Clinical Genetics, Erasmus University, Rotterdam, The Netherlands; 8 Department of Human Genetics, Radboud University Medical Centre Nijmegen, Nijmegen, The Netherlands; 9 Dept. Of Human Genetics, Leiden University Medical Center, Leiden, The Netherlands; 10 Dept. Of Pathology, Leiden University Medical Center, Leiden, The Netherlands; 11 Genetic Epidemiology Unit, IARC and Department of Dermatology, University of Utah, USA; 12 Centre for Biomedical Research in Rare Diseases (CIBER-ER), Madrid, Spain

## Abstract

**Background:**

The recent development of new high-throughput technologies for SNP genotyping has opened the possibility of taking a genome-wide linkage approach to the search for new candidate genes involved in heredity diseases. The two major breast cancer susceptibility genes BRCA1 and BRCA2 are involved in 30% of hereditary breast cancer cases, but the discovery of additional breast cancer predisposition genes for the non-BRCA1/2 breast cancer families has so far been unsuccessful.

**Results:**

In order to evaluate the power improvement provided by using SNP markers in a real situation, we have performed a whole genome screen of 19 non-BRCA1/2 breast cancer families using 4720 genomewide SNPs with Illumina technology (Illumina's Linkage III Panel), with an average distance of 615 Kb/SNP. We identified six regions on chromosomes 2, 3, 4, 7, 11 and 14 as candidates to contain genes involved in breast cancer susceptibility, and additional fine mapping genotyping using microsatellite markers around linkage peaks confirmed five of them, excluding the region on chromosome 3. These results were consistent in analyses that excluded SNPs in high linkage disequilibrium. The results were compared with those obtained previously using a 10 cM microsatellite scan (STR-GWS) and we found lower or not significant linkage signals with STR-GWS data compared to SNP data in all cases.

**Conclusion:**

Our results show the power increase that SNPs can supply in linkage studies.

## Background

Genomewide linkage scans have traditionally been performed using low-density maps of microsatellite markers with a spacing of about 10 cM across the genome [1]. However, the recent development of new high-throughput technologies for SNP genotyping has opened up the possibility of taking a genome-wide approach to study polymorphisms quickly and economically. Moreover, several studies have demonstrated that a map of very closely spaced SNP markers could offer many advantages over the low density maps of microsatellite markers, mainly by increasing the power to detect linkage and consequently more precisely identify the disease locus [2-7]. Although these biallelic markers have lower heterozygosity, they are at a higher density in the genome and they are associated with lower genotyping error rates [8,9]. Additionally, SNP assays are more amenable to multiplexing and are easier to automate, and over 6 million validated human SNPs have been stored in public databases to date.

Genomewide linkage scans have become a widely used tool in the effort to unravel the genetic bases of human hereditary diseases. One example of this is the search for high-penetrance genes involved in breast cancer. The two major breast cancer susceptibility genes, BRCA1 and BRCA2, have been shown to be involved in a significant proportion of families affected with breast and ovarian cancer, but it is clear that about 70% of familial breast cancer is not caused by mutations in these genes [10-13]. These families are known as BRCAX families. Unfortunately, despite intensive efforts, the discovery of additional breast cancer predisposition genes has so far been unsuccessful. [14-16]. Recently, a large linkage scan study of the BCLC (Breast Cancer Linkage Consortium) which included 149 BRCAX families and used traditional microsatellite markers (450 Short Tandem Repeats or STRs) failed to identify any candidate regions that were significant at a genome-wide level [16], presumably because of possible genetic heterogeneity of these families and/or because of recessive/polygenic mechanisms [15,16]. Alternatively, significant linkage may have been missed as a result of sub-optimal coverage of the genome by the STR marker set used, which may not have extracted all the inheritance information contained in the dataset.

Therefore, we have conducted a linkage study with 4.720 SNPs across the genome in nineteen BRCAX families to identify candidate regions containing BRCAX gene(s). We show the existence of different candidate regions linked to a small number of families, and a power improvement by using SNPs instead of microsatellite markers.

## Results

### Candidate regions

**Figure 1:**
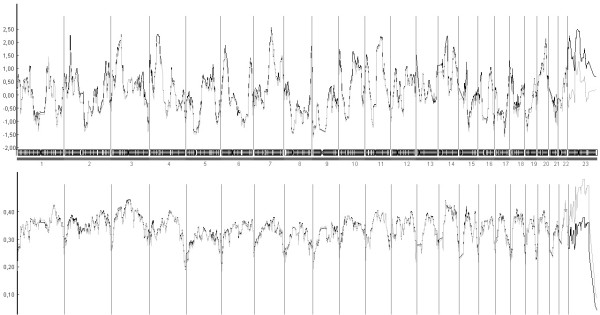
**Multipoint nonparametric linkage analysis of 19 families**. The figure includes LOD scores (top) and information content (bottom) using CEPH and ALL frequencies. Chromosome numbers are indicated below each panel.

Results of multipoint non parametric linkage analysis (NPLA) in all chromosomes for our SNP data (SNP-GWS) are shown in Figure 1 (using ALL frequencies; see methods). Candidate linkage regions were determined as those with an NPLOD score with p-values ≤ 0.01 using CEPH frequencies (see methods), but that were still significant (p-values ≤ 0.05) when more conservative analysis using ALL frequencies was done.

Six regions on six different chromosomes were selected according this criteria to contain susceptibility genes involved in breast cancer using both CEPH and ALL frequencies: chromosome 2, with a maximum NPLOD score of 2.26 and 1.70 (p-values ≤ 0.01 and 0.04 respectively); chromosome 3 with a maximum NPLOD score of 2.29 and 2.19 (p-values ≤ 0.01); chromosome 4 with a maximum NPLOD score of 2.29 and 2.01(p-values ≤ 0.01 and 0.02 respectively); chromosome 7 with a maximum NPLOD score of 2.56 and 2.44 (p-values ≤ 0.01); chromosome 11 with a maximum NPLOD score of 2.21 and 2.15 (p-values ≤ 0.01 and 0.02 respectively) and chromosome 14 with a maximum NPLOD score of 2.25 and 1.89 (p-values ≤ 0.01 and 0.02 respectively. Although the telomeric region of chromosome 8 also fulfilled the criteria to be considered a candidate region, telomeric regions are prone to show inflated LOD score values and this region was therefore not investigated further [see Additional file 1]. In addition, two more regions in chromosome X were identified with NPLOD score with p-values ≤ 0.01 using CEPH frequencies, neither of them were considered because they were not significant when ALL frequencies were applied.

Maximum NPLOD scores and parametric HLOD scores (both dominant and recessive models) for these regions using ALL frequencies are summarized in Table 1.

**Table 1:** Maximum LOD score in candidate regions

**CHR**	**Region**	**From**		**To**		**NPL**	**p value**	**PAR DOM**	**PAR REC**
						
		**SNP**	**cM**	**SNP**	**cM**				
2	2p22.3	rs1054889	56	rs1167465	62				
3	3p21.31p14.3	rs1014228	67	rs920891	70	2.19	0.014	0.74	NL
4	4p14q12	rs1046655	51	rs2538	64	2.01	0.02	1.31	1.12
7	7q21.11q21.3	rs2040902	95	rs722263	105	2.44	0.007	1.02	1.57
11	11q12.3q14.3	rs1525064	67	rs1404532	91	2.15	0.02	1	NL
14	14q13.1q21.3	rs2027338	34	rs1532202	43	1.89	0.03	1	NL

LOD score values under the dominant (PAR DOM), recessive (PAR REC), and nonparametric (NPL) analysis with the *p-values *of the SNP-GWS in 19 BRCAX families using ALL frequencies. NL: no linkage (values < 1 in parametric analysis)

Only a small fraction of families showed significant linkage values (p < 0.05) in these regions when NPLOD score per Family was calculated. Three families were selected in candidate regions in chromosome 3, 4 and 7 with moderate linkage values (from 1.89, p < 0.03, to 5.00 p < 0.00001, in NPL analysis) and a single family with very high NPLOD score value (higher that 5.52 p < 0.00001 to 11.40 p < 0.000001, in NPL analysis) in chromosome 2, 11 and 14 was found (see Table 2). These families with significant NPLOD scores were considered as a linked family in these candidate regions and included in the next fine mapping step using microsatellite markers.

**Table 2:** Maximum NPLOD score values for linkage families in the candidate regions

**CHR**	**FAMILY**	**From**		**To**		**SNP-GWS**	***p value***	**SNP-GWS + STRs**	***p value***
						
		**SNP**	**cM**	**SNP**	**cM**				

2	3395	rs714513	50.2	rs1167465	61.6				

3	154*	rs4796	54.5	rs2030395	68.5	4.07	0.00001	1.90	0.03
3	2191*	rs893367	67.5	rs1392702	70.7	2.06	0.02	0.17	0.4
3	3395*	rs1127732	56	rs1024008	94.5	2.38	0.01	0.64	0.3

4	8	rs936232	37.1	rs894905	60.5	2.22	0.01	2.23	0.01
4	14*	rs12142	49	rs1560605	74.1	3.03	0.001	0.27	0.4
4	2191	rs1456087	39.7	rs1560605	74.1	5.00	0.00001	4.98	0.00001

7	14*	rs917424	88.3	rs875588	164.2	3.42	0.0005	2.84	0.003
7	3386	rs2009526	76.6	rs1990790	134.2	1.89	0.03	1.84	0.03
7	3568	rs917089	98.3	rs322812	131.3	2.33	0.01	2.33	0.01

11	153	rs1675090	66.6	rs586699	92.3	11.40	0.000001	11.47	0.000001

14	153	rs2027338	34.2	rs923908	44	10.50	0.000001	11.53	0.000001

Two different sets of markers are considered: a) SNP-GWS, b) SNP-GWS plus STR fine mapping. *Families ruled out after STR fine mapping.

### Impact of LD on linkage results

**Figure 2:**
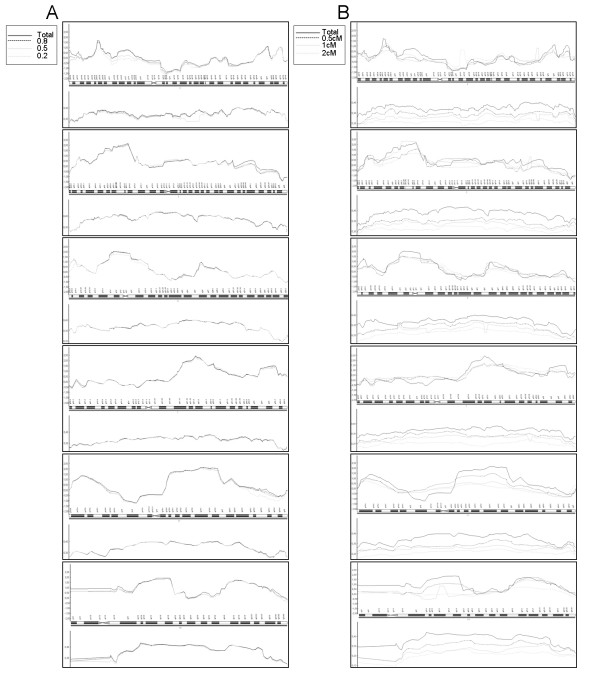
**LOD score values and information content**. LOD score values (top part of each panel) and information content (bottom part of each panel) in the six candidate regions selected, taking into account: A) LD between marker loci (r^2 ^> 0.2/0.5/0.8), and B) marker densities (0.5 cM/1 cM/2 cM).

The inflation of the nonparametric multipoint LOD score due to inter-marker linkage disequilibrium (LD) has been recently described [17,18], and so inter-marker LD should be taken into account in our high-density genome-wide SNP linkage screen. Two analyses were performed to asses whether LD affected linkage results in our data, using two different approaches to dealing with LD. Figure 2A shows the comparison of NPLOD score values in the six candidate regions selected using ALL frequencies, taking into account different measures of LD between marker loci (r^2 ^> 0.2/0.5/0.8); the same regions are shown in Figure 2B using different marker densities (0.5 cM/1 cM/2 cM). The same six regions were still identified when discarding markers correlated with different r^2 ^values (0.2/0.5/0.8), that is, almost all candidate regions are maintained with significant p values (p < 0.05) in all analyses (except chromosome 2, using r^2 ^= 0.5 and 0.2). The other method to discard possible markers in LD was to take the genetic distance between them into consideration. We found a more drastic decrease in the NPLOD scores when this approach was used. The information content (IC) value was also calculated and compared for both analyses. In additional information [see Additional file 2] is summarized the number of markers used in all the analysis after removing the markers. Figure 2 shows that the decrease of this value in all regions is higher when the genetic distance between markers is considered than when LD values are considered [see also Additional file 3 for more details; values for NPLOD score using ALL frequencies, *p-values *and IC in both methods are shown].

### Simulation results

We have calculated how many regions with similar NPLOD scores could be expected by chance, in order to examine the false positive rates in our data. Gene dropping simulations were performed. This analysis replaces our real data with simulated chromosomes, maintaining the original pedigree structure, allele frequencies and recombination fraction, and retaining the original missing data patterns. These datasets are generated under the null hypothesis of no linkage or association to observed phenotypes. Using Merlin software, we generated 1000 random genomewide scan replicates of the data and those regions with an NPLOD score with p-values ≤ 0.01 using CEPH frequencies and were still significant (p-values ≤ 0.05) when more conservative analysis using ALL frequencies was done, were computed (according the criteria applied in our candidate region selection).

The distribution of the number of candidate regions identified according these criteria from 1000 randomly simulated genomewide scans is shown [see Additional file 4]. We observed that only 15 replicate scans showed positive scores greater than or equal to seven (the number of regions originally identified in the real data) [see Additional file 1], giving an empirica *p-value *of 0.015. The results found with the simulated data suggest more allele sharing in our data than would be expected by chance.

### Fine mapping analysis in candidate regions

Wiltshire *et al*. [19] demonstrated that true positive peaks are more prone to increase in LOD score when additional informative meioses are sampled, while false positives are not. Although a peak increase in fine mapping is not a proof that the signal is real, the magnitude of the increase is proportional to the increase in the probability of linkage while a peak decrease is associated with a decrease in the subsequent probability of linkage. In order to confirm or discard our peaks in the candidate regions, we did an additional genotyping in these regions with STRs. A total of 50 further STR markers were analysed in the families that had shown positive model-free LOD score values in the candidate regions. The mean and median distances between STRs across candidate regions were 2.5 cM (2.7 Mb) and 2.2 cM (2.3 Mb) respectively. The corresponding values for the SNPs-STRs combined in the fine mapping were 0.9 cM (1.0 Mb) and 0.6 cM (0.7 Mb) respectively across candidate regions.

Maximum NPLOD score comparison in individual families with and without fine-scale markers in our candidate regions are shown in Table 2. We found that linkage analysis including microsatellite markers across the candidate regions showed differences in the maximum LOD score values, either confirming (2p22.3, 4p14q12 7q21.11–7q21.3, 11q13.5–11q14.3 and 14q21.1–14q21.3 regions) or ruling out (3p21.31–3p14.3 region) our candidate regions. Therefore, rather than narrowing the regions, the fine mapping analysis led to the identification of well-defined positive areas in some regions where the LOD score increases or at the very least remains the same, and ruled out other areas where the LOD values drop. Five of six regions were maintained after the analysis but family 14 was ruled out in the candidate regions on chromosomes 4 and 7. Moreover, the candidate regions on chromosome 3 in all families were ruled out too. A final number of five regions (in chromosome 2, 4, 7, 11 and 14) are proposed as new putative loci to contain susceptibility breast cancer genes.

### SNPs versus microsatellites comparison

Firstly, the data quality was extremely high with successful genotyping obtained for 98.9% of SNP markers compared to STR call rate of 96.7%.

**Figure 3:**
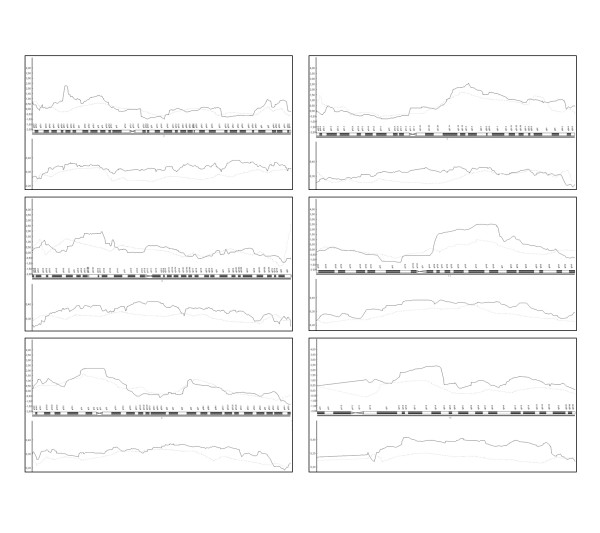
**SNP-GWS *versus *STR-GWS data**. Comparison of SNP-GWS (black) and STR-GWS (grey) results in candidate regions. NPLOD score comparisons are at the top, and information content (IC) comparisons are at the bottom of each panel.

Secondly, in order to evaluate whether a map of closely spaced SNPs offers equal or higher power to detect linkage compared with the traditional approach based on STR-GWS, we compared the linkage values obtained along these six candidate regions. Parametric and nonparametric analyses were performed using 16 of the 19 families with both types of data available, and the nonparametric NPLOD scores obtained with SNP-GWS and STR-GWS are shown in Figure 3 (using ALL frequencies estimating by averaging over all typed individuals). A comparison of information content is also shown in Figure 3.

In all cases, the lowest NPLOD score values were found when microsatellite markers were analyzed. When STR markers were used, the maximum NPLOD scores obtained with SNPs dropped from 2.25 (p = 0.012) to -0.21 (p = 0.6) in chromosome 2, 1.90 (p = 0.03) to 0.42 (p = 0.3) in chromosome 3, 2.22 (p = 0.013) to 1.65 (p = 0.05) in chromosome 4, 2.59 (p = 0.005) to 1.77 (p = 0.04) in chromosome 7 and from 2.51 (p = 0.006) and 2.41 (p = 0.008) to 0.53 (p = 0.3) and 0.98 (p = 0.2) in chromosomes 11 and 14, respectively. Also, our results suggest that SNP mapping allows loci to be defined more precisely than STR marker due to the higher marker density (figure 3).

In order to evaluate the ability of both sets of markers to identify the candidate regions, we used the inclusion criteria of Smith *et al*. [16]: linkage peaks with both non parametric and parametric analysis (NPA and PA) LOD scores greater than 1 for the whole family set (in this case, 16 families), or linkage peak with a parametric LOD score greater that 1.5 in individual families under the Dominant Model (DM). Comparative results by regions under NPA (NPLOD score) and PA (HLOD for dominant and also recessive models) are shown in Table 3. We found that using SNP data (SNP-GWS), all candidate regions showed NPLOD score greater that 1 and HLOD values greater than 1 in all of them except the region in chromosome 3. On the other hand, using STR markers, only three of them would be identified (in chromosome 3, 4 and 7) with NPA and only chromosome 4 had HLOD values greater than 1 for both models when PA was performed. In addition, in Table 4 we summarized those individual families in these regions with parametric HLOD values greater than 1.5 that are considered as linkage families according to Smith *et al*. [16]. Four families were identified using the SNP-GWS set (one in each candidate region) but just two were found when STR-GWS data were used. When high-density STR data (fine-mapping step) were included in the analysis, all the HLOD values were maintained or increased in both SNP-GWS and STR-GWS data. Under this inclusion criterion, Family 3395 in chromosome 2 and Family 153 in chromosome 14 would not have been identified if only STR-GWS had been considered. Moreover, when positive linkage signals using STR-GWS were found, none of them were higher than those obtained with SNP-GWS. These results demonstrate that SNP data would be preferable in order to avoid either suggestive or significant linkage being missed. Finally, when high-density STR data were included in the analysis, all the HLOD values were maintained or increased in both SNP-GWS and STR-GWS data.

**Table 3:** Comparative LOD scores in candidate regions using SNP-GWS and STR-GWS data

		**SNP-GWS**				**STR-GWS**			
**CHR**	**Region**	**NPL**	**p value**	**PAR DOM**	**PAR REC**	**NPL**	**p value**	**PAR DOM**	**PAR REC**

2	2p22.3	**2.25**	0.012	**1.45**	0.20	-0.21	0.600	0.00	0.00
3	3p21.31p14.3	**1.95**	0.030	0.64	0.68	**1.01**	0.200	0.00	0.16
4	4p14q12	**2.22**	0.013	**1.38**	**1.40**	**1.65**	0.050	**1.34**	**1.29**
7	7q21.11q21.3	**2.59**	0.005	**1.00**	**2.01**	**1.77**	0.040	0.48	0.94
11	11q13.5q14.3	**2.51**	0.006	**1.04**	0.20	0.99	0.200	0.84	0.20
14	14q21.1q21.3	**2.41**	0.008	**1.03**	0.30	0.98	0.200	0.75	0.77

Comparative NPLOD scores and PLOD scores (HLOD) under dominant (PAR DOM) and recessive models (PAR REC) using SNP-GWS and STR-GWS data in candidate regions according to Smith et al [15] (NL: values < 1 in NPLOD or PLOD). Values > 1.00 are highlighted in bold.

**Table 4:** Comparative LOD scores in linkage families using SNP-GWS and STR-GWS data both with and without high-density STRs data

		**DM HLOD**		**RM HLOD**	
**CHR**	**Family**	**SNP-GWS**	**SNP-GWS + STRs**	**STR-GWS**	**STR-GWS + STRs**

2	3395	1.73	1.92	NL	NL
4	2191	1.8	1.8	1.74	1.75
11	153	2.2	2.2	1.78	1.98
14	153	2.2	2.2	NL	NL

Comparative parametric LOD scores (HLOD) under dominant and recessive models using SNP-GWS and STR-GWS; both with and without high-density STRs data in linkage families. Candidate families 8, 3386 and 3568 were excluded since none of them had a parametric LOD score greater than 1.5. NL: values < 1.5 in parametric analysis according to Smith et al [15]. SNP-GWS + STRs: SNP-GWS plus STR fine mapping. STR-GWS + STRs: STR-GWS plus STR fine mapping.

## Discussion

The results reported here represent one of the first genomewide scans using SNP markers to identify breast cancer susceptibility loci. We first carried out a SNP-GWS using data from 19 BRCAX families and identified six candidate regions. We used both ALL and CEPH frequencies in this analysis and we found no important differences. This result demonstrates that data analysis using frequencies derived from the data itself is very consistent and robust. We then performed high-density STR mapping with additional microsatellites across identified linkage peaks in an attempt to obtain more convincing support for linkage. We found suggestive evidence of linkage in five of the six candidate regions: 2p22.3, 4p14q12, 7q21.11–7q21.3, 11q13.5–11q14.3 and 14q21.1–14q21.3.

In parallel, simulated data revealed that only 15 in 1000 genomewide scans would have shown at least as many positive LOD score values as we obtained, by chance alone. That is, despite the lack of strong linkage signals in our data, we observed a higher proportion of sharing than would be expected by chance, which suggests that some of our observed candidate peaks could contain susceptibility loci.

We performed parametric (PA) and nonparametric analyses (NPA). Using PA, no exceptionally strong linkage signals were found, since this value depends on the assumed genetic model being correct. This means that evidence for linkage might be missed if we only perform PA. On the other hand, using NPA we identified a region in chromosome 3 that was later discarded when fine mapping genotyping data were considered, but this false positive evidence of linkage was not found when PA was performed. These results highlight the importance of performing both parametric and non-parametric analyses in order to avoid false negative and false positive results.

Regarding our candidate regions, in a previous paper Huusko *et al*. [15] found a linkage signal in 14 Finnish breast cancer families in the 2q32 region D2S2262 (190.8 cM), and another one on chromosome 9, close to D9S283 (93.2 cM). We have not replicated these results in our study which gave evidence at chromosome 2, but in the 2p22.3 region. In addition, a further study has been published including exclusively Swedish non-BRCA1/2 families and using 10.000 SNPs across the genome [20]. This study has reported suggestive linkage (HLOD 2.34) to the 10q23.32–q25.2 region as well as two other loci at 12q14–q21 and 19p13.3–q12, but none of these has been replicated in our study. These results may suggest that these non replicate regions could be specific of both Finnish and Swedish populations respectively. On the other hand, no clear locus conferring a substantial risk was identified in the genome-wide linkage analysis by Smith *et al*. [16], even though the number of families was much higher (149 multiple case breast cancer families). It should be noted that, despite the large number of families included, none of the identified regions meet the classic criteria for significance in linkage for a genomewide linkage study of a LOD score of 3.0. The highest HLOD was 1.8 on chromosome 4 (D4S392; 79 cM). Four other LOD score greater that 1 were found on chromosomes 2 (HLOD 1.21; 17 cM), 5 (HLOD 1.04; 196 cM), 14 (NPL 1.56; 44 cM) and 22 (HLOD 1.15; 41 cM). Obviously, since some families were common in the Smith *et al*. study and in our study, we have also identified the same region on chromosome 4p14q12, but while in Smith *et al*. [16] this result is predominantly due to a single family (FAM2191 in both studies), we have found a second family with evidence of linkage in this region after fine mapping (FAM8). Not surprisingly, SNP analysis also identified a linkage signal in the 11q13.5 region for the family RUL153 that was noted in Smith *et al*. [16]. Similarly we found a linkage signal at position 34–43 cM in chromosome 14 (NPL of 1.89), which is close to the locus (44 cM) previously reported by Smith *et al*. [15]. We also detected a signal at chromosome 2 (HLOD of 1.17), but this was located at position 56 cM, close to marker rs1054889. Smith *et al*. [16] found no signal greater than 1 at our candidate region in chromosome 7.

Smith *et a*l. [16] reported that heterogeneity between families could be a explanation for the lack of evidence of linkage. Polygenic models in which the existence of a large number of breast cancer susceptibility genes, each one conferring modest risk for developing the disease have been also proposed. Recently, Easton *et al*. [21] have performed a genome-wide case-control breast cancer association study that identified five loci containing plausible causative low-risk variants. However none of our candidate regions overlap, probably because linkage approach lacks power to detect alleles with small effects on disease risk. Both scenarios would explain the unsuccessful attempts to identify other breast cancer predisposition genes different from BRCA1 and BRCA2, due to lack of power. Therefore, it seems to be crucial to include in future studies large subsets of families from homogeneous populations, to reduce the genetic variation and moreover increase linkage detection power.

Inflation of the linkage signals may arise especially when intermarker LD is present and pedigree founders are not available. Two analyses were performed to assess whether LD affected the linkage results from our data, each using a different approach to exclude SNPs in order to remove correlated markers. We found that LOD scores were maintained when SNPs were excluded based on observed LD patterns, but when genetic distance was the criterion for exclusion, it resulted in much lower IC values, suggesting that it was too severe. Our results demonstrate that modelling marker-marker LD to eliminate redundant SNPs is sufficient to avoid false positive signals but at the same time limits the decrease in IC and consequent loss of power, and therefore seems to be the best strategy for the optimal use of SNP linkage panels. Besides, the small number of markers discarded in LD modelling analysis indicates that only modest LD was present in the Illumina SNP panel used. Therefore, we can conclude that LD between loci does not significantly affect the overall detection of linkage regions in our SNP genome scan.

We have compared results obtained from SNP markers using the Illumina panel (version III) to those from a traditional 10 cM microsatellite scan, and we have demonstrated that dense SNP maps can provide higher power, identifying regions suggestive of linkage that would be missed by a standard microsatellite scan. Using this strategy, we have observed a clear improvement in the power of linkage signal detection because it is noteworthy that while five regions were consistently identified performing both parametric and non-parametric analysis of SNP-GWS data, only one of these was identified when traditional STR-GWS data were analysed.

## Conclusion

In conclusion, our strategy of two-stage linkage mapping, therefore, has allowed us to identify five new putative loci related to breast cancer with moderate values that suggests the existence of genetic heterogeneity among these non-BRCA1/2 breast cancer families. We also found two regions (11q13.5 and 14q21.1–14q21.3) linked to the same family (FAM153) and confirmed these by fine mapping analysis. This result is consistent with the hypothesis of a polygenic model as has been previously suggested.

Therefore, our results show that genomewide scans using SNP markers, followed by fine mapping using STRs to confirm the veracity of the primary scan, appears to be an optimal strategy for future linkage analyses. In addition, this approach provides more and stronger linkage signals than those using traditional STR markers and allows both to screen for well-defined positive areas in some regions, and to filter any false positive signals.

## Methods

### Families selected

Nineteen families with non-BRCA1/2 hereditary breast cancer from the USA, the Netherlands and Spain were selected for this study. Sixteen of them were previously genotyped using a low density genomewide scan with microsatellite markers and most of them were included in the recent Breast Cancer Linkage Consortium Study [16].

Families had to satisfy the following criteria: a) at least three women diagnosed with breast cancer below age 60 years, b) no case of ovarian cancer or male breast cancer in a blood relative, c) DNA samples available for genotyping from at least three women affected with breast cancer, or from children of affected individuals such that the genotypes might be inferred (in the latter case, at least two children of affected women needed to be available). DNA samples were available from 81 female family members with breast cancer. The nineteen families were recruited by three groups. The Spanish families (5 families) were ascertained by the Familial Cancer Unit at The Spanish National Cancer Centre (CNIO) in Madrid; the American families (7 families) were ascertained by Henry Lynch at Creighton University in Omaha Nebraska and originally genotyped at the International Agency for Cancer Research (IARC); and the Dutch families (7 families) were ascertained by the Clinical Genetic Centres in Leiden and Rotterdam and through the Netherlands Foundation for the Detection of Hereditary Tumours (STOET) [see Additional file 5]. In all cases the DNA of one affected member of each family was studied, and the presence of BRCA1 and BRCA2 gene mutations was ruled out through different methods (sequencing or DHPLC). The presence of large deletions and insertions was analysed using MLPA (Multiplex Ligation Probe Amplification), deletion junction-PCR or Southern analysis.

### Markers and genotyping

SNPs markers were genotyped using the Illumina BeadArray linkage mapping panel (version III). Oligonucleotides were designed and synthesized by Illumina, Inc. Details of the GoldenGate assay have been previously described [22,23]. A total of 4763 genomewide SNPs are included in the panel, with mean and median physical spacing's of 0.6 Mb and 0.4 Mb, respectively. Mean and median genetic spacing's were 1.5 cM and 1.1 cM, respectively [24]. Final markers included in the study are shown [see Additional file 2].

For sixteen of the nineteen families, a total of 400 polymorphic microsatellite or STR markers from the ABI Prism Linkage Mapping Set-MD10 (Applied Biosystems) were analysed on ABI 3700 DNA sequencers at the Welcome Trust Sanger Institute. The average interval between the markers was 10 cM. Genotypes were called automatically with Genotyper software.

In addition, in order to narrow down potential regions of interest based on the analysis of the genomewide SNPs (GWS-SNPs), we selected STRs with high heterozygosity across candidate regions (one every 2–3 cM). Fine density genotyping around linkage peaks (8 STRs/region on the average) was performed in families that showed suggestive linkage. Genotyping was performed at the CNIO using the ABI 3700 DNA sequencer platform and data analysis was carried out using Genescan software.

### Quality control data

To control the quality of experimental variables such as plate orientation, and to provide the opportunity to test genotyping reproducibility, inter- and intra-plate duplicate genotyping was performed. The PEDSTATS software was used to determine the genotyping success rate, to confirm the pedigree structure, and to correctly specify the relationships between individuals in each family [25]. The PEDCHECK program [26] was used to detect marker typing incompatibilities in pedigree data, which can be due to errors in pedigree structure or sample switching. All ambiguous marker genotypes were deleted. On the other hand, the error-detection approach in Merlin [27] was used to search for Mendelian inconsistencies within each of the SNP clusters, discarding genotypes that gave contradictory information about gene flow in a pedigree considering all available data simultaneously and these improbable genotypes were removed using the program's wipe function. The data quality was extremely high, with successful genotyping obtained for 98.9% of SNP markers after removing Mendelian errors and genotyping failures. In contrast, the call rate for STR markers was 96.7%.

### Allele frequencies and genetic maps

The analysis of all families combined was done assuming that all families had the same genetic background, coming from the same homogeneous (European) population. Due to the limited number of families (19 families) and individuals (81 individuals), the allele frequencies were not estimated separately in each of the three populations (Spanish, Dutch and American, with 17 individuals, 30 individuals, and 34 individuals, respectively), nor among the founders (only 17 individuals). We therefore estimated allele frequencies across all individuals pooled. In order to demonstrate that the estimated allele frequencies derived from the full data set (ALL frequencies) were appropriate for the analysis, we compared them with the frequencies provided by Illumina for each marker, estimated from 82 unrelated CEPH individuals (CEPH frequencies). We found no statistically significant differences after Bonferroni corrections (data not shown). Focusing on the highest non parametric LOD score using NPL all statistics (NPLOD score) values identified, we compared the results obtained using both types of frequencies and we found no important differences (the average change was just 0.13) [see Additional file 1].

In order to obtain more conservative results and avoid false positives due to specific population differences, all analyses with SNP markers have been performed using allele frequencies estimated from the full data set.

Frequency estimation for genomewide scan data with microsatellites was carried out according to Smith *et al*. [16]. For the fine-mapping markers in the candidate regions, we genotyped an additional 50 unrelated Spanish controls to obtain better allele frequency estimates. A genetic map using a total of 4,737 loci for each chromosome using 518 meioses derived from 28 CEPH pedigrees were constructed by Illumina [24], and this CEPH recombination map was used for SNP analysis (excluding pseudoautosomal regions). For microsatellite data, we used the STR genetic map constructed by deCODE Genetics Inc [28].

### Statistical Analysis

Multipoint nonparametric linkage analysis (NPL all statistic) for SNP markers was performed using Merlin and MINX (Merlin in X) programs [27,29] to calculate Z means using NPL all statistics (NPLOD score) [30]. For the parametric linkage analysis (PLA) we assumed a dominant model with a susceptibility allele with population frequency 0.003. This model is based on Claus *et al*. [31], where risks were modelled in seven age-categories and implemented in 14 liability classes, with separate classes for unaffected and affected persons [1]. We also considered a recessive model (RM), under which the risks were the same as those under the dominant model (DM), but the susceptibility allele population frequency was 0.08. Multipoint and singlepoint analyses for the whole family set and for each family individually were performed using both CEPH and ALL frequencies, and heterogeneity LOD scores (HLOD) [32] under dominant or recessive models and NPLOD scores were calculated. LOD score for individual families using per Family option in Merlin software was also calculated.

Candidate linkage regions were defined as those with NPLOD scoreswith associated p-values < 0.01 using CEPH frequencies, but that were still significant (p-values < 0.05) when more conservative analysis (using ALL frequencies) was done. The genomewide scan with microsatellite data and additional fine-mapping microsatellite data were also analysed using the Merlin program and parametric and non parametric analyses were performed.

We compared the linkage of a genomewide scan using SNPs alone (SNP-GWS) with a genomewide scan using microsatellites alone (STR-GWS) in sixteen families for which data from both types of scan were available. Parametric and non parametric analyses were performed.

The power of linkage analysis and consequently the expected LOD score is related to the amount of information extracted from the map [33]. The information content (IC) provides a measure of information that can be extracted from pedigree data using a particular marker set as compared to an infinitely dense set of markers with complete genotyping on the entire pedigree. This measure is a function of marker heterozygosity and the completeness of genotyping, and when multipoint analysis is used, IC is also a function of density. Information content for the two marker panels tested (SNP-GWS and STR-GWS) was calculated with Merlin.

The average r^2 ^between adjacent markers across the genetic map was 0.11 ± 0.003 in the Illumina III panel (SNP-GWS), based on 82 CEPH individuals. To determine the effect of LD between marker loci, two approaches were taken. Under the first, SNPs in strong LD with other SNPs (r^2 ^values > 0.2/0.5/0.8) were removed and the data set reanalysed. Under the second, NPLOD scores (ALL frequencies) were calculated using an approach for modelling LD between markers during multipoint analysis [34]. We also performed the comparison using different marker densities (0.5 cM/1 cM/2 cM).

To evaluate our linkage results, we used Merlin's simulation option to empirically estimate the probability that the observed proportion of the genome that showed excess allele sharing could be observed by chance. One thousand genomewide replicates were analysed under the null hypothesis of no linkage to breast cancer, and the percentage of the genome with an NPLOD score over the specified threshold was determined in each genomewide replicate.

## Abbreviations

SNP- Single Nucleotide Polymorphism; 

STR- Short Tandem Repeat; 

LD- Linkage disequilibrium; 

GWS- Genomewide scan; 

NPLOD score- Non parametric LOD score; 

PLA- Parametric linkage analysis; 

NPLA- Non parametric linkage analysis; 

HLOD score- Heterogeneity LOD score; 

DM- Dominant model; 

RM- Recessive model.

## Authors' contributions

EG, JMR, MS carried out the genotyping. AO, JB and AGN participated in the design of the study. AGN, GP, JMR participated performing the statistical analysis. JB, DG and PD have helped to draft the manuscript. MS, OS, HR, RO, CA, NH and JB have provided the samples and DG and PD have provided the microsatellite data to allow the SNP-STR comparisons. All authors read and approved the final manuscript.

## Supplementary Material

Additional file 1 List of candidate regions. List of candidate regions selected and LOD score comparison using full data set (ALL) frequencies and Illumina frequencies (CEPH). Click here for file

Additional file 2 SNP markers used in all the analyses Click here for file

Additional file 3 Values for NPLOD score, IC and *p-values *for both methods used to modelling marker-marker LD. NPLOD score, IC and *p-values *were calculated considering, different measures of LD between marker loci (left table) and, different measures of genetic distance between marker loci (right table) Click here for file

Additional file 4 Number of candidate regions observed from simulated data. Distribution of the number of candidate regions with an NPLOD score with p-values ≤ 0.01 using CEPH frequencies and still significant (p-values ≤ 0.05) when more conservative analysis using ALL frequencies, identified from 1000 times random genomewide scan data. Click here for file

Additional file 5 Summary of families by population group. Click here for file
