# Supplementary material for: Genomewide high-density SNP linkage analysis of non-BRCA1/2 breast cancer families identifies various candidate regions and has greater power than microsatellite studies
Source: BMC Genomics. 2007 Aug 30;8:299. doi: 10.1186/1471-2164-8-299 (PMC2072960; doi:10.1186/1471-2164-8-299)
Supplement: Additional file 1 — List of candidate regions. List of candidate regions selected and LOD score comparison using full data set (ALL) frequencies and Illumina frequencies (CEPH). [file 1471-2164-8-299-S1.doc]

Additional file 1

List of candidate regions

|  |  | **CEPH** | | **ALL** | |
| --- | --- | --- | --- | --- | --- |
| **Chromosome** | **Region** | **NPL(Max)** | ***p value*** | **NPL(Max)** | ***p value*** |
| 2 | 2p22.3 | 2.26 | 0.01 | 1.7 | 0.04 |
| 3 | 3p21.31p14.3 | 2.29 | 0.01 | 2.19 | 0.01 |
| 4 | 4p14q12 | 2.29 | 0.01 | 2.01 | 0.02 |
| 7 | 7q21.2q21.3 | 2.56 | 0.01 | 2.44 | 0.01 |
| 11 | 11q13.5q14.3 | 2.21 | 0.01 | 2.15 | 0.02 |
| 14 | 14q21.1q21.3 | 2.25 | 0.01 | 1.89 | 0.03 |
| 8 | 8ter | 2.31 | 0.01 | 2.11 | 0.02 |

List of candidate regions selected and LOD score comparison using full data set (ALL) frequencies and Illumina frequencies (CEPH).
