# Supplementary material for: Genomewide high-density SNP linkage analysis of non-BRCA1/2 breast cancer families identifies various candidate regions and has greater power than microsatellite studies
Source: BMC Genomics. 2007 Aug 30;8:299. doi: 10.1186/1471-2164-8-299 (PMC2072960; doi:10.1186/1471-2164-8-299)
Supplement: Additional file 2 — SNP markers used in all the analyses [file 1471-2164-8-299-S2.doc]

Additional file 2

SNP markers used in all the analyses.

|  |  |  | **Linkage Disequilibrium Modelling** | | | **Genetic density** | | |
| --- | --- | --- | --- | --- | --- | --- | --- | --- |
| **Chromosome** | **Full SNPs** | **Quality SNPs** | **r^2^=0.8** | **r^2^=0.5** | **r^2^=0.2** | **0.5 cM** | **1 cM** | **2 cM** |
| 1 | 385 | 381 | 363 | 353 | 339 | 183 | 138 | 95 |
| 2 | 394 | 392 | 375 | 355 | 312 | 182 | 135 | 90 |
| 3 | 364 | 359 | 348 | 337 | 305 | 151 | 119 | 77 |
| 4 | 276 | 270 | 265 | 259 | 242 | 124 | 96 | 64 |
| 5 | 276 | 274 | 257 | 246 | 208 | 130 | 101 | 70 |
| 6 | 296 | 292 | 273 | 262 | 239 | 134 | 97 | 59 |
| 7 | 271 | 270 | 258 | 245 | 227 | 129 | 99 | 64 |
| 8 | 212 | 209 | 199 | 192 | 180 | 104 | 77 | 56 |
| 9 | 176 | 171 | 168 | 165 | 154 | 97 | 77 | 53 |
| 10 | 207 | 204 | 198 | 186 | 178 | 101 | 72 | 54 |
| 11 | 186 | 186 | 178 | 173 | 157 | 89 | 70 | 48 |
| 12 | 231 | 228 | 216 | 206 | 187 | 107 | 79 | 57 |
| 13 | 169 | 168 | 162 | 158 | 146 | 79 | 57 | 40 |
| 14 | 173 | 169 | 161 | 155 | 142 | 74 | 54 | 38 |
| 15 | 166 | 165 | 149 | 141 | 128 | 70 | 53 | 38 |
| 16 | 163 | 162 | 157 | 153 | 137 | 72 | 53 | 38 |
| 17 | 132 | 131 | 124 | 116 | 111 | 63 | 50 | 39 |
| 18 | 131 | 131 | 122 | 117 | 112 | 60 | 52 | 39 |
| 19 | 112 | 109 | 99 | 93 | 85 | 52 | 42 | 31 |
| 20 | 118 | 117 | 111 | 103 | 99 | 58 | 46 | 31 |
| 21 | 87 | 87 | 69 | 69 | 57 | 36 | 29 | 21 |
| 22 | 86 | 86 | 83 | 81 | 77 | 41 | 31 | 21 |
| **TOTAL*** | **4611** | **4561** | **4335** | **4165** | **3822** | **2136** | **1627** | **1123** |

* Chromosome X not included.
