# Supplementary material for: Genomewide high-density SNP linkage analysis of non-BRCA1/2 breast cancer families identifies various candidate regions and has greater power than microsatellite studies
Source: BMC Genomics. 2007 Aug 30;8:299. doi: 10.1186/1471-2164-8-299 (PMC2072960; doi:10.1186/1471-2164-8-299)
Supplement: Additional file 3 — Values for NPLOD score, IC and p-values for both methods used to modelling marker-marker LD. NPLOD score, IC and p-values were calculated considering, different measures of LD between marker loci (left table) and, different measures of genetic distance between marker loci (right table) [file 1471-2164-8-299-S3.doc]

Additional file 3

Values for NPLOD score, IC and  *p-values* for both methods used to modelling marker-marker LD

| **Chr** | **r^2^** | **NPL Max** | **IC** | ***pvalue*** |  | **Chr** | **cM** | **NPL Max** | **IC** | ***pvalue*** |
| --- | --- | --- | --- | --- | --- | --- | --- | --- | --- | --- |
| **2** | none | 1.70 | 0.37 | 0.05 |  | **2** | none | 1.70 | 0.37 | 0.05 |
|  | 0.8 | 1.56 | 0.37 | 0.05 |  |  | 0.5 | 0.90 | 0.30 | 0.20 |
|  | 0.5 | 1.50 | 0.36 | 0.06 |  |  | 1 | 1.01 | 0.25 | 0.20 |
|  | 0.2 | 1.49 | 0.36 | 0.06 |  |  | 2 | 0.49 | 0.21 | 0.30 |
| **3** | none | 2.19 | 0.38 | 0.02 |  | **3** | none | 2.19 | 0.38 | 0.02 |
|  | 0.8 | 2.13 | 0.37 | 0.02 |  |  | 0.5 | 1.56 | 0.29 | 0.05 |
|  | 0.5 | 2.08 | 0.37 | 0.02 |  |  | 1 | 2.20 | 0.28 | 0.02 |
|  | 0.2 | 2.04 | 0.37 | 0.02 |  |  | 2 | 1.15 | 0.21 | 0.20 |
| **4** | none | 2.01 | 0.32 | 0.02 |  | **4** | none | 2.01 | 0.31 | 0.02 |
|  | 0.8 | 1.99 | 0.32 | 0.03 |  |  | 0.5 | 1.51 | 0.29 | 0.06 |
|  | 0.5 | 1.92 | 0.31 | 0.03 |  |  | 1 | 1.50 | 0.27 | 0.06 |
|  | 0.2 | 1.74 | 0.31 | 0.05 |  |  | 2 | 1.56 | 0.22 | 0.05 |
| **7** | none | 2.44 | 0.32 | 0.007 |  | **7** | none | 2.44 | 0.32 | 0.007 |
|  | 0.8 | 2.41 | 0.32 | 0.007 |  |  | 0.5 | 1.64 | 0.26 | 0.05 |
|  | 0.5 | 2.35 | 0.32 | 0.01 |  |  | 1 | 1.72 | 0.26 | 0.05 |
|  | 0.2 | 2.29 | 0.33 | 0.01 |  |  | 2 | 1.62 | 0.17 | 0.05 |
| **11** | none | 2.15 | 0.38 | 0.01 |  | **11** | none | 2.15 | 0.38 | 0.01 |
|  | 0.8 | 2.14 | 0.38 | 0.01 |  |  | 0.5 | 1.43 | 0.32 | 0.06 |
|  | 0.5 | 2.09 | 0.38 | 0.02 |  |  | 1 | 0.63 | 0.27 | 0.30 |
|  | 0.2 | 2.04 | 0.38 | 0.02 |  |  | 2 | 0.19 | 0.23 | 0.50 |
| **14** | none | 1.89 | 0.41 | 0.03 |  | **14** | none | 1.86 | 0.42 | 0.03 |
|  | 0.8 | 1.89 | 0.41 | 0.03 |  |  | 0.5 | 1.03 | 0.33 | 0.12 |
|  | 0.5 | 2.00 | 0.41 | 0.02 |  |  | 1 | -0.21 | 0.25 | 0.60 |
|  | 0.2 | 1.96 | 0.41 | 0.03 |  |  | 2 | -0.003 | 0.25 | 0.50 |

NPLOD score, IC and  *p-values* were calculated considering, different measures of LD between marker loci (left table) and, different measures of genetic distance between marker loci (right table)
