# Supplementary material for: Genomewide high-density SNP linkage analysis of non-BRCA1/2 breast cancer families identifies various candidate regions and has greater power than microsatellite studies
Source: BMC Genomics. 2007 Aug 30;8:299. doi: 10.1186/1471-2164-8-299 (PMC2072960; doi:10.1186/1471-2164-8-299)
Supplement: Additional file 4 — Number of candidate regions observed from simulated data. Distribution of the number of candidate regions with an NPLOD score with p-values ≤ 0.01 using CEPH frequencies and still significant (p-values ≤ 0.05) when more conservative analysis using ALL frequencies, identified from 1000 times random genomewide scan data. [file 1471-2164-8-299-S4.doc]

Additional file 4

Number of candidate regions observed from simulated data

Distribution of the number of candidate regions with an NPLOD score with p-values ≤ 0.01 using CEPH frequencies and still significant (p-values ≤ 0.05) when more conservative analysis using ALL frequencies, identified from 1000 times random genomewide scan data.
