# Supplementary material for: Genomewide high-density SNP linkage analysis of non-BRCA1/2 breast cancer families identifies various candidate regions and has greater power than microsatellite studies
Source: BMC Genomics. 2007 Aug 30;8:299. doi: 10.1186/1471-2164-8-299 (PMC2072960; doi:10.1186/1471-2164-8-299)
Supplement: Additional file 5 — Summary of families by population group. [file 1471-2164-8-299-S5.doc]

Additional file 5

Summary of families by population group.

| **Country** | **Families** | **Total samples** | **Total affected** | **Families w. 3 affected** | **Families w. > 3 affected** |
| --- | --- | --- | --- | --- | --- |
| Spain | 5 | 17 | 15 | 5 | 0 |
| France | 7 | 34 | 27 | 3 | 4 |
| Netherlands | 7 | 30 | 30 | 0 | 7 |
| Total | 19 | 81 | 72 | 8 | 11 |
